# BBCAnalyzer: a visual approach to facilitate variant calling

**DOI:** 10.1186/s12859-017-1549-4

**Published:** 2017-02-28

**Authors:** Sarah Sandmann, Aniek O. de Graaf, Martin Dugas

**Affiliations:** 1 Institute of Medical Informatics, University of Münster, Albert-Schweitzer-Campus 1, Münster, 48149 Germany; 2 Laboratory Hematology, RadboudUMC, Geert Grooteplein zuid 8, Nijmegen, 6525 Netherlands

**Keywords:** Variant calling, Next-generation sequencing, Visualization, Personalized medicine, Hotspot mutations

## Abstract

**Background:**

Deriving valid variant calling results from raw next-generation sequencing data is a particularly challenging task, especially with respect to clinical diagnostics and personalized medicine. However, when using classic variant calling software, the user usually obtains nothing more than a list of variants that pass the corresponding caller’s internal filters. Any expected mutations (e.g. hotspot mutations), that have not been called by the software, need to be investigated manually.

**Results:**

BBCAnalyzer (Bases By CIGAR Analyzer) provides a novel visual approach to facilitate this step of time-consuming, manual inspection of common mutation sites. BBCAnalyzer is able to visualize base counts at predefined positions or regions in any sequence alignment data that are available as BAM files. Thereby, the tool provides a straightforward solution for evaluating any list of expected mutations like hotspot mutations, or even whole regions of interest. In addition to an ordinary textual report, BBCAnalyzer reports highly customizable plots. Information on the counted number of bases, the reference bases, known mutations or polymorphisms, called mutations and base qualities is summarized in a single plot. By uniting this information in a graphical way, the user may easily decide on a variant being present or not – completely independent of any internal filters or frequency thresholds.

**Conclusions:**

BBCAnalyzer provides a unique, novel approach to facilitate variant calling where classical tools frequently fail to call. The R package is freely available at http://bioconductor.org. The local web application is available at Additional file 2. A documentation of the R package (Additional file 1) as well as the web application (Additional file 2) with detailed descriptions, examples of all input- and output elements, exemplary code as well as exemplary data are included. A video demonstrates the exemplary usage of the local web application (Additional file 3).

Additional file 3: Supplement_3. Video demonstrating the exemplary usage of the web application “BBCAnalyzer”. (MP4 11571 kb)

**Electronic supplementary material:**

The online version of this article (doi:10.1186/s12859-017-1549-4) contains supplementary material, which is available to authorized users.

## Background

Personalized medicine is striving to find its way into clinical routine. Some therapeutic decisions are already influenced by the presence or absence of certain mutations. Sequencing in such contexts is usually performed using next-generation sequencing (NGS). However, the analysis of NGS data may be complicated by a number of reasons. On the one hand, false positive calls due to artifacts in the sequencing data may harm the results. On the other hand, false negative calls due to low variant allele frequencies or bad coverage of certain regions can lead to true mutations not being detected.

There are many of programs in terms of variant calling, like GATK [1] or SAMtools [2]. However, – apart from some user-definable thresholds – these programs are usually a black box. They take a BAM file as input, apply a number of partially complex filtration steps and return a VCF file containing a list of likely variants. Variants with low allelic frequencies – even if they are known hotspot mutations – are usually excluded to keep the number of false positive calls low. Even recently developed sophisticated approaches like MutAid [3] that provide a complete data analysis pipeline for Sanger- and next-generation sequencing data, including different mapping and variant calling algorithms, finally report a VCF file per sample and a variant summary table. Furthermore, tools developed for NGS analysis in a clinical setting, e.g. CSN and CAVA [4], usually focus on the correct annotation of previously called variants.

To our knowledge, there are no freely available variant calling tool that can use as input a list of likely mutations that should be considered with an especially low allelic frequency. Neither is it possible to perform a comparable analysis of positions where no variant is called, but the user is interested in the raw number of bases. As a consequence, a manual, time-consuming inspection of all sites where mutations are expected but not called is necessary.

The integrative genomics viewer (IGV) [5] provides such an option for the manual investigation of the different number of bases, deletions and insertions at any position in the genome. However, it is time consuming to load different samples into the program and to look at the different positions of interest. Moreover, there is no way of automatically summing up and visualizing the base counts for a list of positions in IGV. Other tools, like the R packages Rsamtools [6] and VariantTools [7], provide options for counting the number of bases and determining quality summaries at a selected set of positions. However, as the output is textual, programming one’s own solution for visualization is necessary. Altogether, this does not appear to be a satisfactory solution for clinical routine.

In the use case of medical diagnostics, a tool performing detailed analyses of those locations where mutations are expected – but not always called – may prove to be very useful. Low allelic frequency and bad base quality may often explain why calls are missed. However, in case of missing calls this information is not included in a VCF file and is difficult to obtain from IGV.

To facilitate the analysis of called and uncalled mutations, we developed BBCAnalyzer (Bases By CIGAR Analyzer). Just like common variant callers, the application uses a BAM file as input. It analyzes the CIGAR strings that characterize the mapped reads and reports the number of bases, deletions and insertions at any predefined position or region in comparison to any reference genome in a novel visual- and a common textual way. The analysis as well as the plots may be customized in a number of ways.

## Implementation

BBCAnalyzer is implemented in R and available at http://bioconductor.org. Additionally, a web application for BBCAnalyzer is available at Additional file 2. The tool works with any sequence alignment data that are available as BAM files.

### Input

In addition to the BAM files to be analyzed, BBCAnalyzer needs the corresponding BAI files. When using BBCAnalyzer as a classical R package, a file summing up the sample names and a target file containing the positions or regions to be analyzed have to be provided. When using BBCAnalyzer as a web application, the sample names and the target regions to be analyzed have to be provided in an input field. Optionally, a VCF file containing known mutations or polymorphisms and VCF files for each sample resulting from earlier performed variant calling may be provided.

### Preparation of the plotting

Prior to the actual plotting, BBCAnalyzer performs various steps of preparation.

First, an analysis of the target region is performed. BBCAnalyzer is able to deal with target regions as well as single target bases.

Next, detected bases, deletions and insertions at every defined position are written out by analyzing available CIGAR strings. Furthermore, the quality for every base (also insertions) is written out. A threshold for excluding reads with bad mapping quality may be defined. BBCAnalyzer equally copes with uncovered positions as well as insertions >1 bp.

Subsequently, the number of detected bases, deletions and insertions at every position is summed up. Additionally, mean quality of detected bases – including insertions and for the inserted bases only – is calculated. A threshold may be set to exclude bases with bad quality. The number of excluded bases is counted and reported separately. In case of VCF files being available for every sample, the expected number of bases, deletions and insertions are determined based on the called alternate alleles and the assigned genotypes.

Finally, the ratios of the detected bases, deletions and insertions (additionally) are determined. Based on the ratios, up to six different calls are reported. A threshold may be set to ignore variants with a minor ratio. In the case of an available VCF file containing the called variants for every sample, the call – taking into account the reference allele, the alternate alleles and the genotype – is determined.

### Plotting

**Fig. 1:**
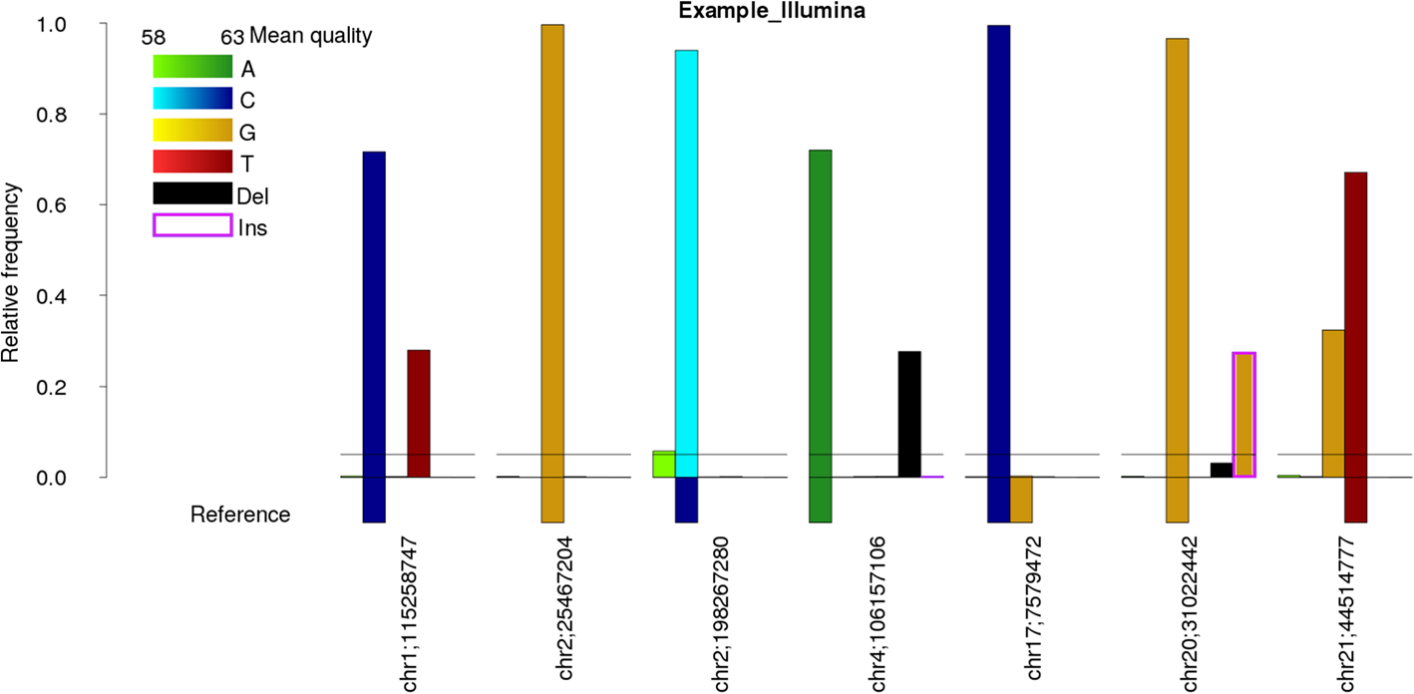
Exemplary output file from real patient data generated by Illumina NextSeq. Relative number of reads at *seven positions* analyzed in case of sample “Example_Illumina”. Reference bases are *plotted* at the *negative y axis*, detected bases in the mapped reads are *plotted* at the *positive y axis* (marked 5% threshold). Likely SNV at chr1:115,258,747 (reference C, ∼70% of the reads with high-quality C and ∼30% of the reads with high-quality T). No variant at chr2:25,467,204 (reference G, ∼100% of the reads with high-quality G). Unlikely SNV at chr2:198,267,280 (reference C, ∼95% of the reads with low-quality C, ∼5% of the reads with low-quality A). Likely deletion at chr4:106,157,106 (reference A, ∼75% of the reads with high quality A, ∼25% of the reads with deleted A). Known homozygous SNP at chr17:7,579,472 (reference G, polymorphism C displayed as additional reference base, ∼100% of the reads with high-quality C). Possible insertion of a “G”, but unlikely deletion at chr20:31,022,442 (reference G, ∼97% of the reads with high-quality G, ∼3% of the reads with deleted G, ∼30% of the reads with inserted high-quality G). Likely SNV at chr21:44,514,777 (reference T, ∼65% of the reads with high-quality T, ∼35% of the reads with high-quality G)

The essential feature of BBCAnalyzer is its plotting functionality. On the basis of the determined base counts, bar plots are generated. The user can choose between one bar plot per sample, focusing the analysis on various target bases at a time, or one bar plot per position, setting the focus of the analysis on the direct comparison of many BAM files at a single position. In addition, the absolute or relative number of counts can be plotted (see Fig. 1).

In each case, the previously determined numbers of detected bases, deletions and insertions are plotted on the positive y axis of the bar plot. The bars are colored according to the base (adenine: green; cytosine: blue; guanine: yellow; thymine: red; deletion: black; insertion: color of the inserted base or bases plus purple edge). If the mean quality of the bases is considered, a lower and an upper boundary may be set. A high mean quality close to the upper bound leads to a darker coloring of the bar.

In addition to the counted bases, the reference bases (hg19 in Fig. 1) at the targeted positions are plotted on the negative y axis. In doing so, single nucleotide variants (SNVs) may directly be identified (Fig. 1, chr1:115,258,747 or chr21:44,514,777 in comparison to chr2:198,267,280).

If a VCF file containing known variants, e.g. originating from dbSNP [8], is given, additional reference bases are included in the plots at the corresponding positions (Fig. 1, chr17:7,579,472).

BBCAnalyzer is able to visualize insertions of 1 bp (Fig. 1, chr20:31,022,442), but also long insertions of > 1 bp by repeatedly analyzing the same position. This is even possible, when many samples are analyzed in parallel, but the corresponding position is not covered in all cases. Furthermore, BBCAnalyzer is able to cope with different inserted bases at the same position by plotting them as stacked bars.

An exemplary visualization of a deletion is displayed at position chr4:106,157,106 in Fig. 1.

If VCF files for all samples are provided, the expected number of bases, deletions and insertions on the basis of the assigned genotype are added to the bar plots using dashed lines.

To account for the ratio of detected bases, deletions and insertions – even if their absolute numbers are chosen to be plotted –, a vector may be defined giving the ratios at which horizontal lines shall be drawn in the plots, e.g. 25, 50 and 75%. However, this function may also be used to mark a variant calling threshold at e.g. 5% (Fig. 1).

### Output

The bases and their qualities at every analyzed position, the absolute number of counts and the relative frequencies together with the corresponding calls get reported as separate TXT files.

When using BBCAnalyzer as an R package, the user can provide an output directory for the plots. If he does, all plots generated by BBCAnalyzer get automatically saved as PNG files. Otherwise, the plots are returned to the workspace.

When using BBCAnalyzer as a web application, all plots generated by BBCAnalyzer get automatically saved as PNG files in the given output directory. Additionally, all plots get displayed in the application’s output panel.

## Results and discussion

The performance of every variant calling tool is influenced by numerous parameters. General characteristics of the data, e.g. average coverage or the level of sequencing errors, can prevent a true mutation from being called, but variant-specific characteristics, e.g. the variant allele frequency or the surrounding bases may also influence mutation calling. Calling mutations in repetitive regions as well as low-frequency mutations is especially challenging.

With the help of two exemplary cases of real sequencing data (Illumina NextSeq) from patients with myelodysplastic syndrome (MDS), we show how BBCAnalyzer is able to facilitate variant calling where common variant calling tools frequently fail.

### Using BBCAnalyzer to detect mutations in repetitive regions

Depending on the repeated motive, calling mutations in repetitive regions can be very challenging. As an example, we consider the insertion of G in case of a homopolymeric stretch of eight Gs (chr20:31022442;insG). This mutation in ASXL1 is regarded as a hotspot mutation for MDS and other myeloid malignancies.

We perform variant calling with two standard tools: GATK and SAMtools. Five sets of sequencing data are analyzed (UPN1-UPN5). Only in case of UPN1 the expected mutation on ASXL1 is called. Furthermore, the mutation is only called by GATK, not by SAMtools. GATK reports a depth of *DP*=188. 142 reads containing the reference allele and 43 reads containing the alternate allele are reported. Thus, the allelic frequency is *VAF*=0.23. The presence of the mutation in UPN1 was confirmed by Sanger sequencing [9].

**Fig. 2:**
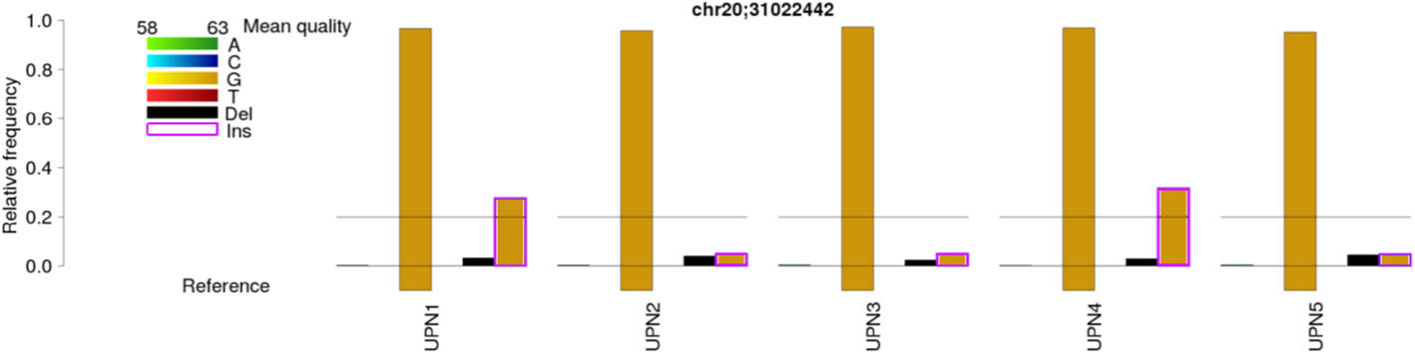
Analysis of position chr20:31,022,442 with BBCAnalyzer. Relative number of reads (*one bar plot* per position; marked 20% threshold): UPN1 and UPN4 feature an inserted G in almost 30% of the reads, while samples UPN2, UPN3 and UPN5 feature no significant difference between the number of reads containing a deletion and the number of reads containing an insertion. Thus, only samples UPN1 and UPN4 are likely to feature the mutation chr20:31,022,441 A >AG

In addition to classical variant calling, we use BBCAnalyzer to analyze position chr20:31,022,442. The results are displayed in Fig. 2. The following options were defined: no vcf input for every sample, no file containing known mutations, reference genome hg19, MQ threshold=60, BQ threshold=50 (ASCII coded; corresponds to Phred quality score of 17), frequency threshold=0.01, quality lower bound=58 (ASCII coded; corresponds to Phred quality score of 25), quality upper bound=63 (ASCII coded; corresponds to Phred quality score of 30), mark at 20%, plot relative number of bases, return one plot per analyzed position.

The analysis of position chr20:31,022,442 with BBCAnalyzer underlines our previous findings in case of sample UPN1. More than 20% of the reads feature an inserted G. However, the frequencies determined by BBCAnalyzer differ considerable from the ones reported by GATK. 4855 reads contain the reference allele, 10 reads contain an A, 159 a deletion, 1394 an insertion and 56 are excluded due to low base quality. Obviously, GATK applies a number of filters by default that exclude a majority of calls.

Considering the remaining sequencing data, it becomes obvious that samples UPN2, UPN3 and UPN5 feature no significant difference between the number of reads containing a deletion and the number of reads containing an insertion. It is thus likely, that these samples do not have a true mutation at the analyzed position. Sanger sequencing also indicated that the mutation was not present in these samples. Sample UPN4 however shows results that are very similar compared to UPN1. 12,355 reads contain the reference allele, 21 reads contain an A, 2 reads contain a C, 1 read contains a T, 370 reads contain a deletion, 4051 reads contain an insertion and 183 reads are excluded due to low base quality. Thus, the allelic frequency of the insertion is *VAF*=0.32. Again we perform Sanger sequencing and indeed the presence of the mutation was confirmed, although it has neither been called by GATK, nor by SAMtools.

### Using BBCAnalyzer to detect low-frequency mutations

Detecting low-frequency mutations in NGS data is, even if data features a low level of sequencing errors, very challenging. Some mutations, like chr1:115,258,744;C >A on NRAS, are important hotspot mutations that occur in various types of cancer at various VAFs. NRAS mutations may occur at low frequency and seem to be associated with transformation to more aggressive disease. Therefore, it may be relevant to detect these mutations already at a subclonal level. For a common variant calling tool, like GATK or SAMtools, it is practically impossible to detect such a variant.

Based on the real Illumina NextSeq sequencing data we simulate five sets of sequencing data using ART_Illumina 2.5.8 [10]. To account for specific sequencing errors, we determine a read quality profile on the basis of the real Illumina data and use it for the subsequent simulation. Average coverage of the simulated samples corresponds to average coverage of the real samples (4034x). Using bam surgeon [11] we simulate the hotspot mutation in NRAS in case of three out of five samples with an expected allelic frequency of *VAF*=0.03.

**Fig. 3:**
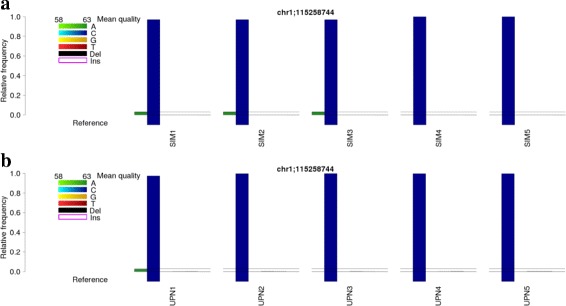
Analysis of position chr1:115,258,744 with BBCAnalyzer. Relative number of reads (*one bar plot* per position; marked 3% threshold): **a**: Simulated data. A low-frequency mutation (C >A) can be observed in case of samples SIM1, SIM2 and SIM3, but not in samples SIM4 and SIM5. **b**: Real data. Similar to the simulated data, the same low-frequency mutation can be observed in case of sample UPN1, but not in samples UPN2-UPN5. Thus, samples UPN1 is likely to feature the mutation chr1:115,258,744 C >A

We perform variant calling on our simulated samples using GATK and SAMtools. However, both tools are unable to detect the mutation we simulated in any of the three samples. In addition to classical variant calling, we again perform analysis of position chr1:115,258,744 using BBCAnalyzer. The results of the simulated samples are displayed in Fig. 3A. The following options were defined: no vcf input for every sample, no file containing known mutations, reference genome hg19, MQ threshold=60, BQ threshold=50 (ASCII coded; corresponds to Phred quality score of 17), frequency threshold=0.01, quality lower bound=58 (ASCII coded; corresponds to Phred quality score of 25), quality upper bound=63 (ASCII coded; corresponds to Phred quality score of 30), mark at 3%, plot relative number of bases, return one plot per analyzed position.

The plot shows that the hotspot mutation on NRAS is clearly visible in case of SIM1, SIM2 and SIM3. The observed frequency matches our expectations. Furthermore, it can be observed that the base quality of both, reads featuring the reference- and reads featuring the alternate allele, is high.

Just like in case of the simulated data, we analyze five sets of real Illumina NextSeq sequencing data. Again, GATK and SAMtools do not report a mutation at chr1:115,258,744 in case of any of the analyzed samples. However, when using BBCAnalyze, the mutation is visible in case of UPN1 (see Fig. 3B). The allelic frequency is *VAF*=0.02. Altogether, BBCAnalyzer detects 10,048 reads containing the reference allele, 247 reads containing an A, 12 reads containing a T, 14 reads containing a deletion and 231 reads being excluded due to low base quality. However, similar to the simulated data, reads featuring the reference- and reads featuring the alternate allele have a high base quality. It thus appears likely, that sample UPN1 actually contains the NRAS mutation.

As there is no way of validating mutations at such a low allelic frequency using Sanger sequencing, we sequenced the sample a second time on Illumina NextSeq (completely separate experiment with target enrichment, library preparation and sequencing). The same mutation can be observed with a similar frequency. Therefore, we assume that sample UPN1 actually features the low-frequency hotspot mutation in NRAS, although GATK and SAMtools both fail to call it.

## Conclusions

Different from common variant calling tools, we present an application that provides a novel, visual approach to facilitate variant calling. Information on the counted number of bases, the reference bases, known mutations or polymorphisms, called mutations and base qualities is united in a novel graphical way. Thereby, BBCAnalyzer provides a solution for otherwise time-consuming, manual inspection of sites where mutations are expected but not called. Considering examples of mutations in repetitive regions and low-frequency mutations, we have shown how BBCAnalyzer can facilitate variant calling where classical tools frequently fail. Additionally, BBCAnalyzer is not only available as a classical R package, but also in form of an intuitively usable web application. This enables a potentially wide usage of our tool – not only by bioinformaticians, but also biologists and clinicians.

## Additional files

Additional file 1 Supplement_1. Detailed documentation of the R package “BBCAnalyzer”. (PDF 377 kb)

Additional file 2 Supplement_2. Scripts and documentation of the web application “BBCAnalyzer”. (ZIP 709 kb)

## Abbreviations

IGV: Integrative genomics viewer
MDS: Myelodysplastic syndrome
NGS: Next-generation sequencing
SNV: Single nucleotide variant
