# Supplementary material for: BBCAnalyzer: a visual approach to facilitate variant calling
Source: BMC Bioinformatics. 2017 Feb 28;18:133. doi: 10.1186/s12859-017-1549-4 (PMC5330023; doi:10.1186/s12859-017-1549-4)
Supplement: Additional file 1 — Supplement_1. Detailed documentation of the R package “BBCAnalyzer”. (PDF 377 kb) [file 12859_2017_1549_MOESM1_ESM.pdf]

BBCAnalyzer: A Visual Approach to Facilitate Variant Calling

Documentation of the R/Biocoductor package: BBCAnalyzer

1 Introduction

The analysis of alignment data is an essential step in the process of analyzing sequencing data. Mutations like single nucleotide base changes, deletions and insertions may be identified in this step.

Usually, the available programs – like GATK [1] or SAMtools [2] – take a bam file, containing the alignment data in a compressed form, as input and return a vcf file containing the called variants and a selection of parameters characterizing them. The overall depth as well as the allele frequencies are returned as two of these parameters.

However, the programs do usually not report the unfiltered number of reads covering a certain mutated base, but they apply a set of partially complex filtration steps. Thresholds – internally or user-defined – are usually used to reject additional variants with a minor ratio. Furthermore, to our knowledge it is not possible to perform a comparable analysis of positions where no variant is called.

The integrative genomics viewer (IGV) [3] provides a possibility for investigating the different number of bases, deletions and insertions at any position in the genome. Yet, it is time consuming to load different samples into the program and to look at the different positions of interest. Moreover, there is no way of automatically summing up and visualizing the base counts in IGV.

With regards to the comparison of different sequencing techniques, it appears useful to have a tool that is able to visualize the background at a selection of locations where e.g. one technique calls a variant but another technique does not. Furthermore, it seems helpful to have a possibility for quickly analyzing positions of expected mutations, which have not been called.

This package provides a possibility for visualizing the number of counted bases, deletions and insertions at any given position in any genome in comparison to the reference bases. Additionally,

- markers for the relative base frequencies,
- the mean qualities of the detected bases,
- known mutations or polymorphisms (e.g. based on dbSNP [4]) and
- called variants in the data

may equally be included into the plots.

1.1 Loading the package

The package can be downloaded and installed with

```
> biocLite("BBCAnalyzer")
```

After installation, the package can be loaded into R by typing

```
> library(BBCAnalyzer)
```

into the R console.

BBCAnalyzer requires the R-packages *VariantAnnotation*, *Rsamtools* and *BSgenome.Hsapiens.UCSC.hg19*. All of them are loaded automatically when using *BBCAnalyzer*.

2 Analyzing the bases and visualizing the results

BBCAnalyzer performs an analysis of the bases, deletions and inserts at defined positions in sequence alignment data and visualizes the results. The process consists of different steps. These steps are highly dependent on each others output, which is why it is necessary to run the function `analyzeBases`, performing the whole process of analyzing the data and visualizing the results, at first. If any changes concerning the plotting of the results are desired, the function `analyzeBasesPlotOnly` may be used, taking amongst others the return value of `analyzeBases` as an obligatory input.

2.1 Preparation

For the correct functioning of *BBCAnalyzer* in the first place, various input files are necessary:

- The names of the samples to be analyzed have to be provided by a file. There has to be one sample name per line without the ".bam"-suffix.

Examplary object *samples*:

```
> sample_file <- system.file("extdata", "SampleNames.txt", package = "BBCAnalyzer")
> samples <- read.table(sample_file)
> samples

                 V1
1 Example_IonTorrent
2        Example_454
```

- The bam- and the corresponding bai files of the samples to be analyzed have to be provided in a folder. The names of the files have to match the names provided by the sample names file.

Example files (*Example_IonTorrent.bam*, *Example_IonTorrent.bai*, *Example_454.bam* and *Example_454.bai*).

- The target regions have to be provided by a file. The file may either contain regions (chromosome, tab, first base of a region, tab, last base of a region) or positions (chromosome, tab, position). A mixture of both is not supported. Yet, a region may cover only one base, i.e. the first and last base of a region may be identical.

Examplary object *targetRegions*:

```
> target_file <- system.file("extdata", "targetRegions.txt", package = "BBCAnalyzer")
> targetRegions <- read.table(target_file)
> targetRegions
```

```
  V1        V2
1  4 106157698
2 20  31024054
3 21  36206893
4  2  25469913
5 21  44514970
6  7 148543694
7  7 148543695
```

Additionally to the necessary input, optional input may be defined:

- If vcf files shall be considered, the corresponding files of the samples to be analyzed have to be provided in a folder. There has to be one file per sample. The names of the files have to match the names provided by the sample names file.

Selected parts of the file *Example_IonTorrent.vcf*:

```
> vcf_file <- system.file("extdata", "Example_IonTorrent.vcf", package = "BBCAnalyzer")
> vcf_IT <- read.table(vcf_file)
> vcf_IT
```

```
  V1       V2 V3 V4  V5      V6   V7
1 21 44514969  .  A ATG 1961.73 PASS

1 AC=1;AF=0.500;AN=2;BaseQRankSum=-1.103;ClippingRankSum=-0.061;DP=190;EFF=INTRAGENIC(MODIFIER||||M
              V9                           V10
1 GT:AD:DP:GQ:PL 0/1:84,92:176:99:1999,0,1942
```

- A tabix file containing e.g. known polymorphisms or mutations (dbSNP [4]) may be provided.

2.2 Analyzing the bases

The analysis of the bases, which actually only serves as a preparation for the final plots, is performed in different steps:

1) Determine target

- If the target regions file contains regions to be analyzed, the different positions covered by the regions are determined.
- If the file already contains single positions, the program directly proceeds with the next step.
- It is not necessary that the regions or positions are ordered.

- If a known insert is supposed to be analyzed, the position of the base succeeding the insert has to be given.

2) Analyze reads

- The reads at every targeted position get analyzed. By the help of the CIGAR string the bases, deletions and inserts are determined. The output is saved as [Sample].bases.txt.
- For every base – also the inserted ones – the base quality is determined ("-1" in case of a deletion). The output is saved as [Sample].quality.txt.
- Reads with a mapping quality below a user defined mapping quality threshold get excluded from the analysis. Instead of a base, "MQ" is noted in the base file. Instead of a quality value, "-2" is noted in the quality file.
- The function copes with uncovered positions ("NotCovered" in the base- and the quality file) and insertions >1bp (repeated analysis of the position).

3) Analyze frequency

- The number of detected bases, deletions and inserts at every position is summed up. Additionally, the mean quality of the detected bases – including the inserts and for the inserted bases only – is calculated.
- Bases with a quality below a user defined base quality threshold are excluded and counted separately.
- The output is saved as [Sample].frequency.txt.
- If the analysis shall consider vcf files as well, the alternate alleles and the genotypes – as far as they are available for the positions analyzed – are written out as well. Furthermore, for every called variant it is noted whether it is an insert.
- The function copes with up to two different alternate alleles per position.

4) Report variants

- The ratios of the detected bases, deletions and insertions (additionally) at every position are determined. According to the determined ratios, up to six different calls get reported. If the user defines a frequency threshold, minor variants with ratios below this threshold do not get reported. The output is saved as [Sample].calling.txt.
- The function copes with insertions >1bp even if the position at which an insert is detected is not covered by all samples being analyzed.
- If the analysis shall consider vcf files as well, the call – taking the reference allele, the alternate allele(s) and the genotype into account – is written out as well. For every called variant it is noted whether it is an insert and whether the genotype is heterozygous.

A list of lists is returned containing all the information that is generated in this analysis step. The list with the bases ([Sample].bases.txt) is available as the first element of the list. The corresponding qualities ([Sample].qualities.txt) are available as the second element of the list. The summed up number of bases ([Sample].frequency.txt) are available as the third element of the list. The calculated relative frequencies and the potential calls ([Sample].calling.txt) are available as the fourth element.

```
> #Example1
> library("BSgenome.Hsapiens.UCSC.hg19")
> ref_genome<-BSgenome.Hsapiens.UCSC.hg19
> output1<-analyzeBases(sample_names=sample_file,
+             bam_input=system.file("extdata",package="BBCAnalyzer"),
+             target_regions=target_file,
+             vcf_input="",
+             output=system.file("extdata",package="BBCAnalyzer"),
+             output_pictures="",
+             known_file=system.file("extdata","dbsnp_138.part.vcf.gz",package="BBCAnalyzer"),
+             genome=ref_genome,
+             MQ_threshold=60,
+             BQ_threshold=50,
+             frequency_threshold=0.01,
+             qual_lower_bound=58,
+             qual_upper_bound=63,
+             marks=c(0.01),
+             relative=TRUE,
+             per_sample=TRUE)
```

First lines of the output file *Example_IonTorrent.bases.txt*

```
> head(output1[[1]][[1]])

  4;106157698 20;31024054 21;36206893 2;25469913 21;44514970 21;44514970.1 7;148543694 7;148543695
1           C           A           G          T           T             T           A           A
2           T           A           G          C           T             T           A           A
3           T           A           G          C           T             T           A           A
4           T           A           A          C           T             T           A           A
5           T           A           G          C        I, T          I, G           A           A
6           T           A           G          C           T             T           A           A
```

First lines of the output file *Example_IonTorrent.quality.txt*

```
> head(output1[[2]][[1]])

  4;106157698 20;31024054 21;36206893 2;25469913 21;44514970 21;44514970.1 7;148543694 7;148543695
1          66          66          66         66          66            66          66          66
2          66          66          66         66          66            66          66          66
3          66          66          66         66          66            66          66          66
```

```
4           66          66          66          66          66            66          66          66
5           66          66          66          66          66            66          66          66
6           66          66          66          66          66            66          66          66
```

First lines of the output file *Example_454.bases.txt*

```
> head(output1[[1]][[2]])
```

```
  4;106157698 20;31024054 21;36206893 2;25469913 21;44514970 7;148543694 7;148543695
1           T           A           G          T  NotCovered           D           D
2           T           G           G          T        <NA>           D           A
3           C           G           G          C        <NA>           D           A
4           T           G           G          C        <NA>           D           D
5           C           A           G          C        <NA>           D           D
6           T           G           G          C        <NA>           D           D
```

First lines of the output file *Example_454.quality.txt*

```
> head(output1[[2]][[2]])
```

```
  4;106157698 20;31024054 21;36206893 2;25469913 21;44514970 7;148543694 7;148543695
1          66          59          66         66  NotCovered          -1          -1
2          66          58          66         66        <NA>          -1          66
3          66          58          66         66        <NA>          -1          66
4          66          58          66         66        <NA>          -1          -1
5          66          56          66         66        <NA>          -1          -1
6          66          58          66         66        <NA>          -1          -1
```

First lines of the output file *Example_IonTorrent.frequency.txt*

```
> head(output1[[3]][[1]])
```

```
    chr       pos ref   a   a_qual   c   c_qual   g   g_qual   t   t_qual Del Ins Ins_a Ins_a_qual
1  chr4 106157698   T  NA       NA 128 61.00000  NA       NA 164 61.25000   2  NA    NA         NA
2 chr20  31024054   A 760 60.49605  NA       NA  NA       NA  NA       NA  NA  NA    NA         NA
3 chr21  36206893   G  50 64.24000  NA       NA 282 62.98227  NA       NA  NA  NA    NA         NA
4  chr2  25469913   C  NA       NA 274 64.81752  NA       NA  83 64.30120  NA  NA    NA         NA
5 chr21  44514970   T  NA       NA  NA       NA  NA       NA 268 62.14925   1 122    NA         NA
6 chr21  44514970   T  NA       NA  NA       NA 153 66.00000 146 61.95890   1 153    NA         NA
  Ins_c Ins_c_qual Ins_g Ins_g_qual Ins_t Ins_t_qual excluded
1    NA         NA    NA         NA    NA         NA       50
2    NA         NA    NA         NA    NA         NA       25
3    NA         NA    NA         NA    NA         NA       32
4    NA         NA    NA         NA    NA         NA       18
5    NA         NA    NA         NA   122   62.37705       48
6    NA         NA   153         66    NA         NA       17
```

First lines of the output file *Example_454.frequency.txt*

```
> head(output1[[3]][[2]])
```

```
    chr       pos ref   a    a_qual   c   c_qual   g    g_qual   t    t_qual Del Ins Ins_a Ins_a_qual
1  chr4 106157698   T  NA        NA 383 60.68668  NA        NA 580 61.44483  NA  18    NA         NA
2 chr20  31024054   A 181 63.10497  NA        NA  70 63.20000  NA        NA  NA  NA    NA         NA
3 chr21  36206893   G 121 62.78512  NA        NA 646 58.66099  NA        NA  22  NA    NA         NA
4  chr2  25469913   C  NA        NA 109 64.44954  NA        NA 115 64.68696  NA  NA    NA         NA
5 chr21  44514970   T  NA        NA  NA        NA  NA        NA  NA        NA  NA  NA    NA         NA
6  chr7 148543694   A  16 58.43750  NA        NA  NA        NA  NA        NA 439   1     1         53
  Ins_c Ins_c_qual Ins_g Ins_g_qual Ins_t Ins_t_qual excluded
1    18   56.27778    NA         NA    NA         NA       70
2    NA         NA    NA         NA    NA         NA       NA
3    NA         NA    NA         NA    NA         NA       NA
4    NA         NA    NA         NA    NA         NA        1
5    NA         NA    NA         NA    NA         NA       NA
6    NA         NA    NA         NA    NA         NA       NA
```

First lines of the output file *Example_IonTorrent.calling.txt*

```
> head(output1[[4]][[1]])
```

```
    chr       pos ref    a_freq    c_freq    g_freq    t_freq    del_freq  ins_freq call1 call2
1  chr4 106157698   T        NA 0.4353741        NA 0.5578231 0.006802721        NA     T     C
2 chr20  31024054   A 1.0000000        NA        NA        NA          NA        NA     A  <NA>
3 chr21  36206893   G 0.1506024        NA 0.8493976        NA          NA        NA     G     A
4  chr2  25469913   C        NA 0.7675070        NA 0.2324930          NA        NA     C     T
5 chr21  44514970   T        NA        NA        NA 0.9962825 0.003717472 0.4535316     T   Ins
6 chr21  44514970   T        NA        NA 0.5100000 0.4866667 0.003333333 0.5100000     G   Ins
  call3 call4 call5 call6
1  <NA>    NA    NA    NA
2  <NA>    NA    NA    NA
3  <NA>    NA    NA    NA
4  <NA>    NA    NA    NA
5  <NA>    NA    NA    NA
6     T    NA    NA    NA
```

First lines of the output file *Example_454.calling.txt*

```
> head(output1[[4]][[2]])
```

```
    chr       pos ref    a_freq    c_freq    g_freq    t_freq  del_freq   ins_freq call1 call2
1  chr4 106157698   T        NA 0.3977155        NA 0.6022845        NA 0.01869159     T     C
2 chr20  31024054   A 0.7211155        NA 0.2788845        NA        NA         NA     A     G
3 chr21  36206893   G 0.1533587        NA 0.8187579        NA 0.0278834         NA     G     A
4  chr2  25469913   C        NA 0.4866071        NA 0.5133929        NA         NA     T     C
5 chr21  44514970   T        NA        NA        NA        NA        NA         NA  <NA>  <NA>
6 chr21  44514970   T        NA        NA        NA        NA        NA         NA  <NA>  <NA>
  call3 call4 call5 call6
1   Ins    NA    NA    NA
2  <NA>    NA    NA    NA
```

```
3   Del    NA    NA    NA
4  <NA>    NA    NA    NA
5  <NA>    NA    NA    NA
6  <NA>    NA    NA    NA
```

2.3 Visualizing the results

The function – which may be called separately as well using the function `analyzeBasesPlotOnly` – provides different possibilities for visualizing the results:

- The absolute or the relative number of detected bases, deletions and inserts may be plotted
- One plot per sample or one plot per targeted position may be output. The files are either saved as [Sample].png or chr[number];[position].png.

Apart from these differences, all plots share some general characteristics:

- The function always produces barplots.
- The bars are colored according to the base at the corresponding position (adenine: green; cytosine: blue; guanine: yellow; thymine: red; deletion: black; insertion: lilac edge).
- The color may additionally contain information on the mean quality of the base if bounds for the lower- and upper mean quality are set. In this case, the color of the bars is darker if the mean quality of the base is closer compared to the upper quality bound. The color of the bar is lighter if the mean quality of the base is closer compared to the lower quality bound.
- The reference bases are plotted on the negative y-axis below each position.
- A tabix file containing known variants or mutations may be provided. In this case, more than one reference base is plotted at the corresponding position.
- Every targeted position is labelled according to the chromosome and the position.
- For each position to be analyzed, lines may be drawn representing relative frequencies (defined by user).
- The function copes with different inserted bases at one position (stacked bars) and inserts>1bp, even if these are not covered by all samples.
- If the analysis shall consider vcf files as well, the expected number of detected bases, deletions and inserts – according to the vcf file – is added to the plot using dashed lines.

Exemple: No vcf files, use known polymorphisms (dbSNP), mark 1% threshold, plot relative frequencies, plot per sample

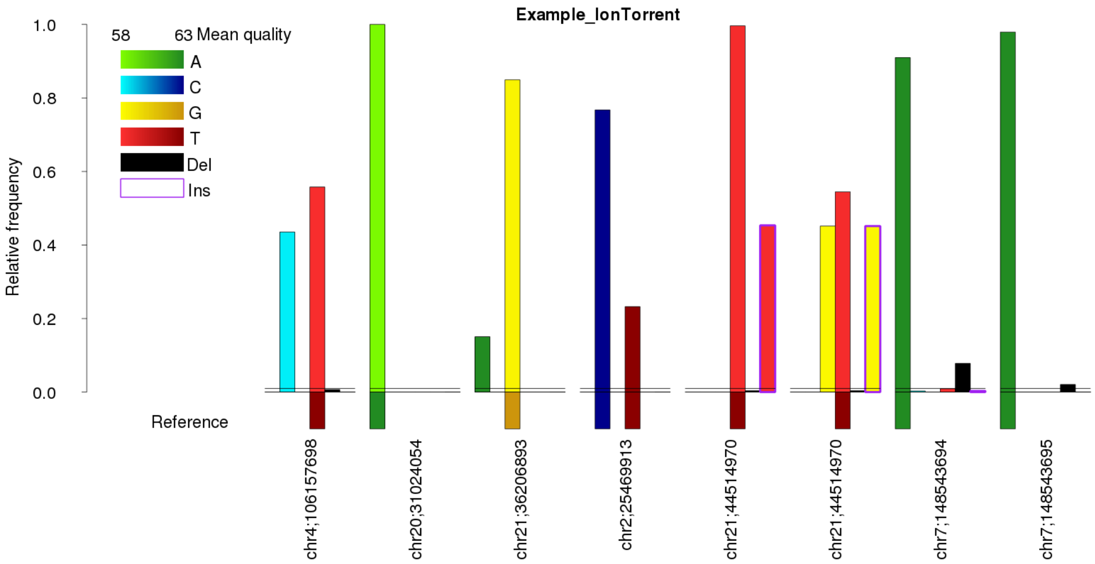

Figure 2.1: Output file Example_IonTorrent.png: Validated SNV at chr4;106157698 (only little number of deleted bases), reference base at chr20;31024054, possible SNV at chr21;36206893, known SNP at chr2;25469913; inserted TG at chr21;44514970, possible deletion at chr7;148543694, possible deletion at chr7;148543695; marked 1% threshold.

Exemple: Use vcf files, use known polymorphisms (dbSNP), mark 20%, 40%, 60%, 80% and 100%, plot number of reads, plot per target.

```
> analyzeBasesPlotOnly(sample_names=sample_file,
+             vcf_input=system.file("extdata",package="BBCAnalyzer"),
+             output="",
+             known_file=system.file("extdata","dbsnp_138.part.vcf.gz",package="BBCAnalyzer"),
+             output_list=output_list,
+             qual_lower_bound=58,
+             qual_upper_bound=63,
+             marks=c(0.2,0.4,0.6,0.8,1),
+             relative=FALSE,
+             per_sample=FALSE)
```

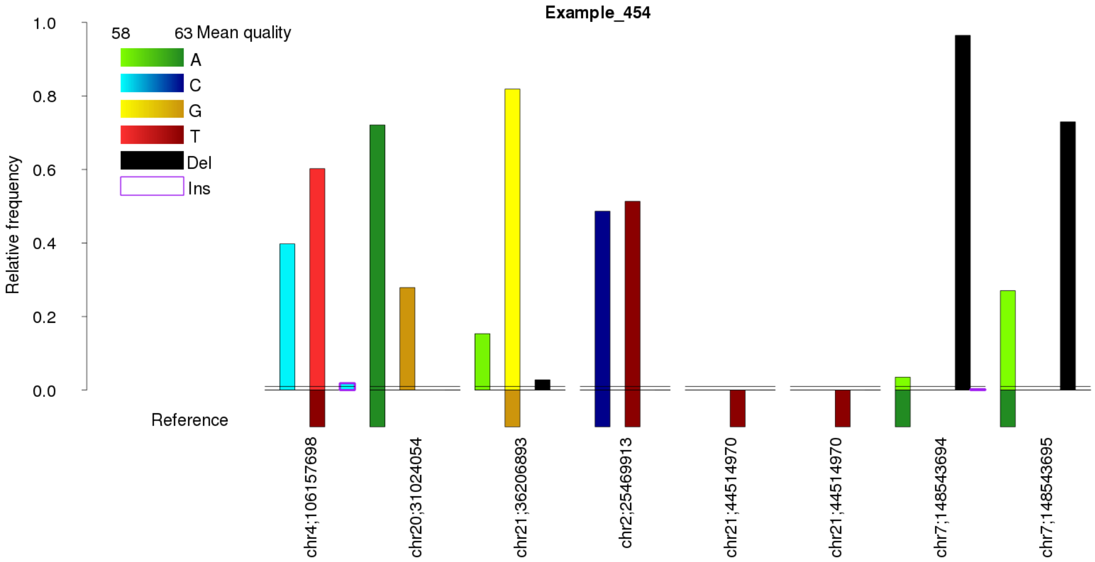

Figure 2.2: Output file Example_454.png: Validated SNV at chr4;106157698 (only a low frequency of inserted A's), likely SNV at chr20;31024054 (high quality of alternate allele G), possible SNV at chr21;36206893 (low quality and frequency of alternate allele A), known SNP at chr2;25469913; not covered position at chr21;44514970, likely deletion at chr7;148543694, likely deletion at chr7;148543695; marked 1% threshold.

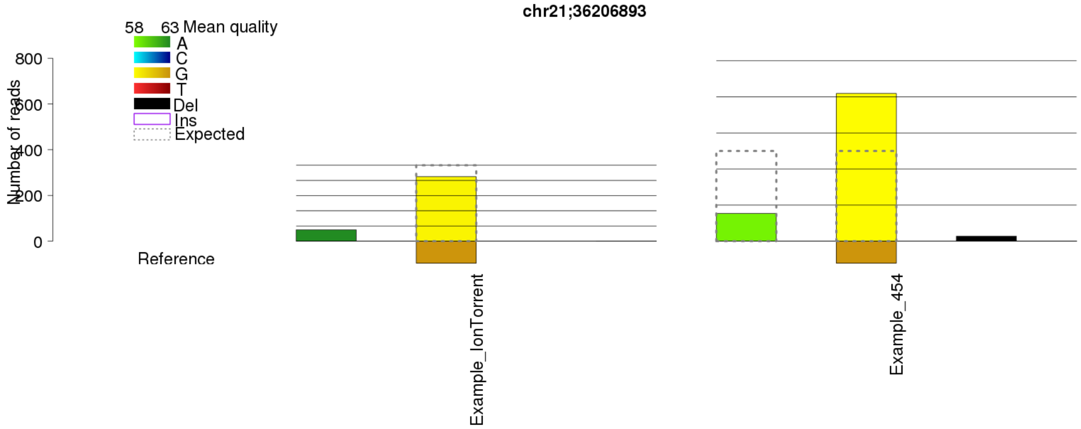

Figure 2.3: Output file chr21;36206893.png: possible SNV according to Example_IonTorrent (not called, homozygous); likely SNV according to Example_454 (called, heterozygous, great deviation from expected number of reads); marked 20%, 40%, 60%, 80% and 100%.

3 Runtime

The runtime of *BBCAnalyzer* depends on the number of samples and the number of positions that get analyzed. Furthermore, the number of reads at the positions being analyzed influences the

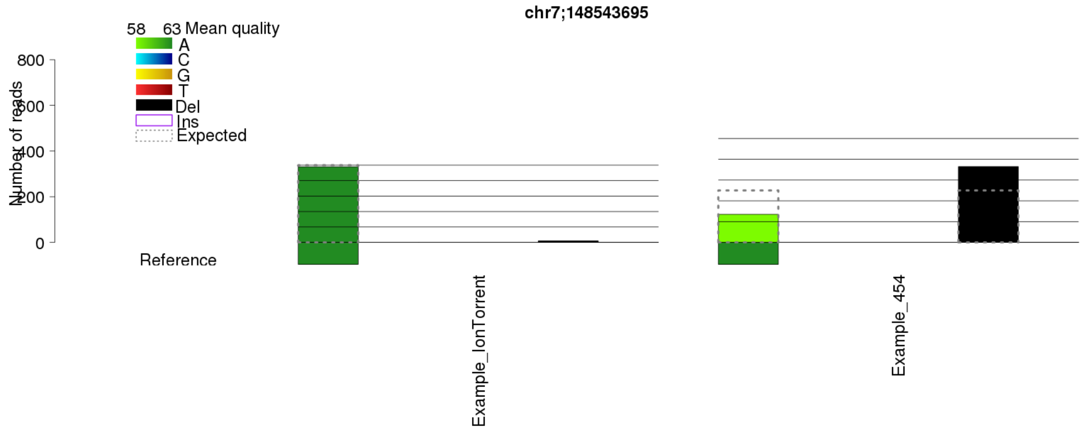

Figure 2.4: Output file chr7;148543695.png: unlikely deletion according to Example_IonTorrent (not called, homozygous); likely deletion according to Example_454 (called, heterozygous, deviation from expected number of reads); marked 20%, 40%, 60%, 80% and 100%.

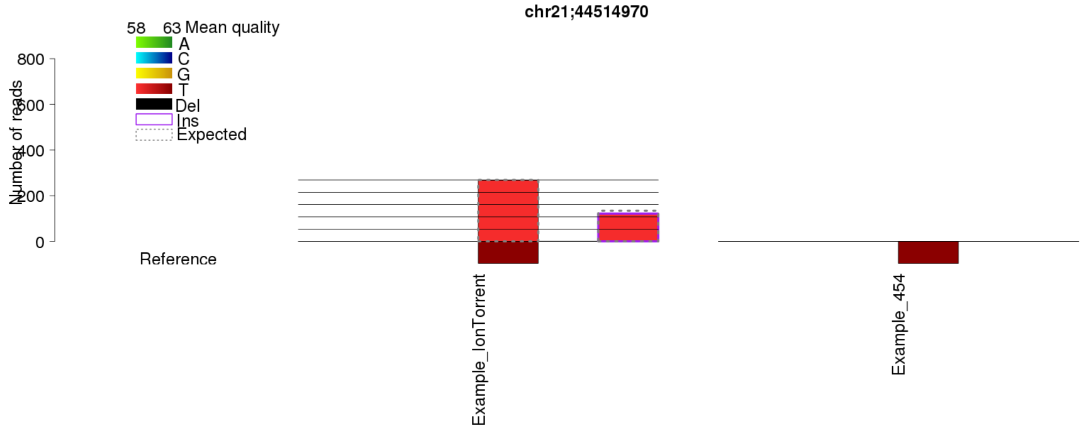

Figure 2.5: Output file chr21;44514970.png: likely insertion according to Example_IonTorrent (called, heterozygous, small deviation from expected number of reads); not covered position in Example_454; marked 20%, 40%, 60%, 80% and 100%.

runtime as well.

The following test case analyzes one position for one sample. 344 reads cover the position being analyzed. A vcf file and a file containing known polymorphisms is not considered (vcf file: increases the runtime in this example by 1.625 seconds; file containing known polymorphisms: increases runtime in this example by 2.273 seconds; both: increases runtime in this example by 2.690 seconds).

Test file *SampleNames_small.txt*:

```
Example_IonTorrent
```

Test files *Example_IonTorrent_small.bam* and *Example_IonTorrent_small.bai*.

Test file *targetRegions_small.txt*:

4 106157698

One barplot per sample is created. The relative frequencies are plotted. The whole pipeline (`analyzeBases`) consumes 40.496 seconds. The plotting itself (`analyzeBasesPlotOnly`) consumes 0.109 seconds.

Exemplary call:

```
##Example 3:
#No vcf files, no file containing known polymorphisms,
mark 1% threshold, plot relative frequencies, plot per sample.
```

```
> output<-analyzeBases(
+             sample_names=system.file("extdata","SampleNames_small.txt",package="BBCAnalyzer"),
+             bam_input=system.file("extdata",package="BBCAnalyzer"),
+             target_regions=system.file("extdata","targetRegions_small.txt",package="BBCAnalyzer"),
+             vcf_input="",
+             output=system.file("extdata",package="BBCAnalyzer"),
+             output_pictures=system.file("extdata",package="BBCAnalyzer"),
+             known_file="",
+             genome=ref_genome,
+             MQ_threshold=60,
+             BQ_threshold=50,
+             frequency_threshold=0.01,
+             qual_lower_bound=58,
+             qual_upper_bound=63,
+             marks=c(0.01),
+             relative=TRUE,
+             per_sample=TRUE)
```

Exemplary output:

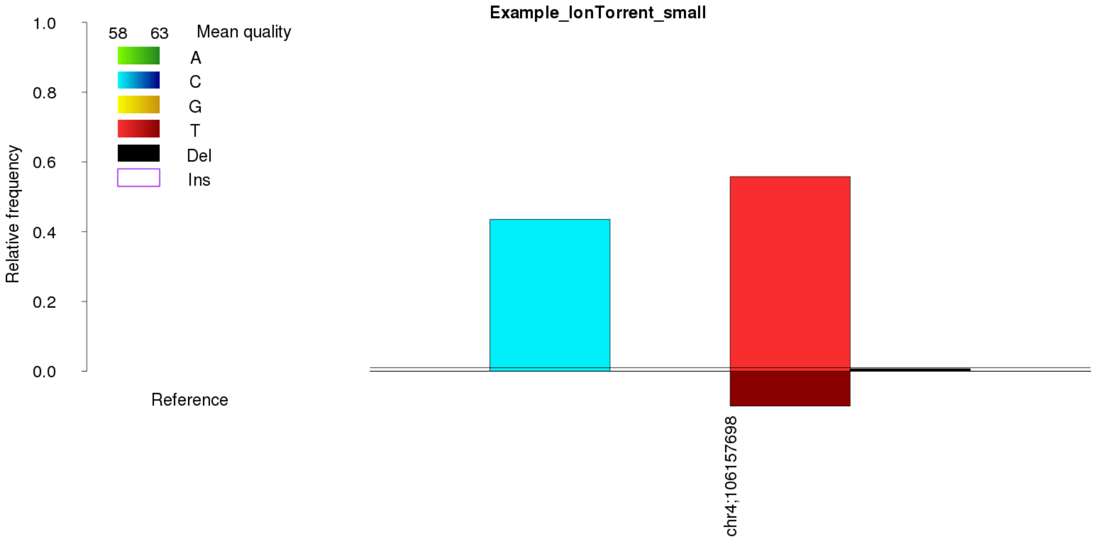

Figure 3.1: Output file Example_IonTorrent_small.png: Validated SNV at chr4;106157698, relative frequencies suggest heterozygous mutation, relatively low mean quality of detected cytosines and adenines ; marked 1% threshold.

References

[1] M DePristo, E Banks, R Poplin, K Garimella, J Maguire, C Hartl, A Philippakis, G del Angel, MA Rivas, M Hanna, A McKenna, T Fennell, A Kernytsky, A Sivachenko, K Cibulskis, S Gabriel, D Altshuler, and M Daly. A framework for variation discovery and genotyping using next-generation dna sequencing data. *Nature Genetics*, 43:491–498, 2011.

[2] H Li, B Handsaker, A Wysoker, T Fennell, J Ruan, N Homer, G Marth, G Abecasis, R Durbin, and 1000 Genome Project Data Processing Subgroup (2009). The sequence alignment/map (sam) format and samtools. *Bioinformatics*, 25:2078–9. PMID: 19505943.

[3] JT Robinson, H Thorvaldsdóttir, W Winckler, M Guttman, ES Lander, G Getz, and JP Mesirov. Integrative genomics viewer. *Nature Biotechnology*, 29:24–26, 2011.

[4] ST Sherry, MH Ward, M Kholodov, J Baker, L Phan, EM Smigielski, and K Sirotkin. dbSNP: the NCBI database of genetic variation. *Nucleic Acids Research*, 29(1), 2001.
