# Supplementary material for: BBCAnalyzer: a visual approach to facilitate variant calling
Source: BMC Bioinformatics. 2017 Feb 28;18:133. doi: 10.1186/s12859-017-1549-4 (PMC5330023; doi:10.1186/s12859-017-1549-4)
Supplement: Additional file 2 — Supplement_2. Scripts and documentation of the web application “BBCAnalyzer”. (ZIP 709 kb) [file 12859_2017_1549_MOESM2_ESM.zip › BBCAnalyzer_local/Documentation.pdf]

BBCAnalyzer: A Visual Approach to Facilitate Variant Calling

Documentation of the web application: BBCAnalyzer_local

1 Left panel

The left panel corresponds to the R/Bioconductor package *BBCAnalyzer*-function "analyzeBases()". It performs a complete analysis of the defined samples and target regions with BBCAnalyzer. The output (txt- and png-files) is saved in the defined output directory. Additionally, the application's progress as well as the generated plots are displayed in the right panel.

Samples to analyze	Names of the samples to analyze (one entry per line, no file name extensions).
Define folder containing bam- and bai-files	Input folder containing the alignment data of all samples to analyze. For each sample defined in "Samples to analyze" there must be a bam- and a bai-file with matching names in the defined folder. Important: Windows-User have to define the folder using double-backslashes (e.g. C:\\home\\BBCAnalyzer_local\\Scripts\\).
Target regions to analyze	Target regions to analyze. Single positions (chromosome and position) as well as longer regions (chromosome, start, end) are equally supported.
Define folder containing vcf files (optional)	Input folder containing vcf files for all samples to analyze. For each sample defined in "Samples to analyze" there must be a vcf-file with matching names in the defined folder.
Define output folder	Folder where output files shall be saved
Define tabix file containing known variants (optional)	Tabix file (format: file.vcf.gz.tbi) containing known variants. For the correct functioning of this option the file "file.vcf.gz" is equally necessary (same folder as "file.vcf.gz.tbi"; does not have to be defined anywhere).
Select reference genome for analysis	Select one of the available reference genomes for analysis. If a genome has not yet been installed, it is automatically downloaded and installed (this process can take a few minutes). Default: BSgenome.Hsapiens.UCSC.hg19.
Mapping quality threshold	A PHRED-scaled value to be used as a mapping quality threshold. All reads with a mapping quality below this threshold are excluded from analysis. Every base in an excluded read gets marked in the output. Default: 60.
Base quality threshold	A PHRED-scaled value to be used as a base quality threshold. All bases with a base quality below this threshold are excluded from analysis. Every excluded base gets marked in the output. The num-

	ber of excluded bases per position gets counted and reported. Default: 50.
Frequency threshold for variant reporting	A frequency to be used as a threshold for variants to be reported. Default: 0.01.
Lower- and upper mean quality bound for color-coding	The lower- and upper bound for the mean quality that shall be color-coded in the plots. All bases with a mean quality below the lower bound are colored with the lightest color defined for the corresponding base. All bases with a mean quality above the upper bound are colored with the darkest color defined for the corresponding base. If the bases shall not be color-coded according to their mean quality, the definable range has to be zero. Default: 58-63.
Select levels at which marks shall be drawn	Levels (relative number of reads) at which horizontal lines shall be drawn in the plots.
Plot number of reads	Relative or absolute number of reads can be plotted. Default: Relative.
Create one plot per	One plot per sample or one plot per position can be created. Default: Sample.

2 Middle panel

The middle panel corresponds to the R/Bioconductor package *BBCAnalyzer*-function "analyzeBasesPlotOnly()". It creates the plots for the defined samples and the previously analyzed target regions with BBCAnalyzer. "Create plots only" is only possible, if a complete analysis of the defined samples with BBCAnalyzer has already been performed and the samples share at least one targeted base that has been analyzed with BBCAnalyzer.

Creating plots with "Create plots only" is usually considerably faster than performing a complete analysis with BBCAnalyzer. Its main function is to change the previously created plots (e.g. change from relative to absolute number of reads or apply different quality bounds for color-coding). However, the function may also be used to combine the output of samples that have been analyzed separately so far.

The new plots are saved in the defined output directory. Additionally, the application's progress as well as the generated plots are displayed in the right panel.

Samples to analyze	Names of the samples to analyze (one entry per line, no file name extensions).
Consider vcf file information (only possible if evaluated in complete analysis)	If vcf file information was available for every sample analyzed with BBCAnalyzer and it was considered in the previously performed complete analysis, it can also be considered for "Create plots only". Default: No.
Define output folder	Folder where previously created output files are stored (sampleX.frequency.txt and sampleX.calling.txt) and new plots shall be saved.

Define tabix file containing known variants (optional)	Tabix file (format: file.vcf.gz.tbi) containing known variants. For the correct functioning of this option the file “file.vcf.gz” is equally necessary (same folder as “file.vcf.gz.tbi”; does not have to be defined anywhere).
Lower- and upper mean quality bound for color-coding	The lower- and upper bound for the mean quality that shall be color-coded in the plots. All bases with a mean quality below the lower bound are colored with the lightest color defined for the corresponding base. All bases with a mean quality above the upper bound are colored with the darkest color defined for the corresponding base. If the bases shall not be color-coded according to their mean quality, the definable range has to be zero. Default: 58-63.
Select levels at which marks shall be drawn	Levels (relative number of reads) at which horizontal lines shall be drawn in the plots.
Plot number of reads	Relative or absolute number of reads can be plotted. Default: Relative.
Create one plot per	One plot per sample or one plot per position can be created. Default: Sample.

3 Right panel

The right panel corresponds to the output screen. The progress as well as problems in the analysis with BBCAnalyzer are displayed. Furthermore, when the analysis is complete, the created plots are displayed as well.

4 Screenshots

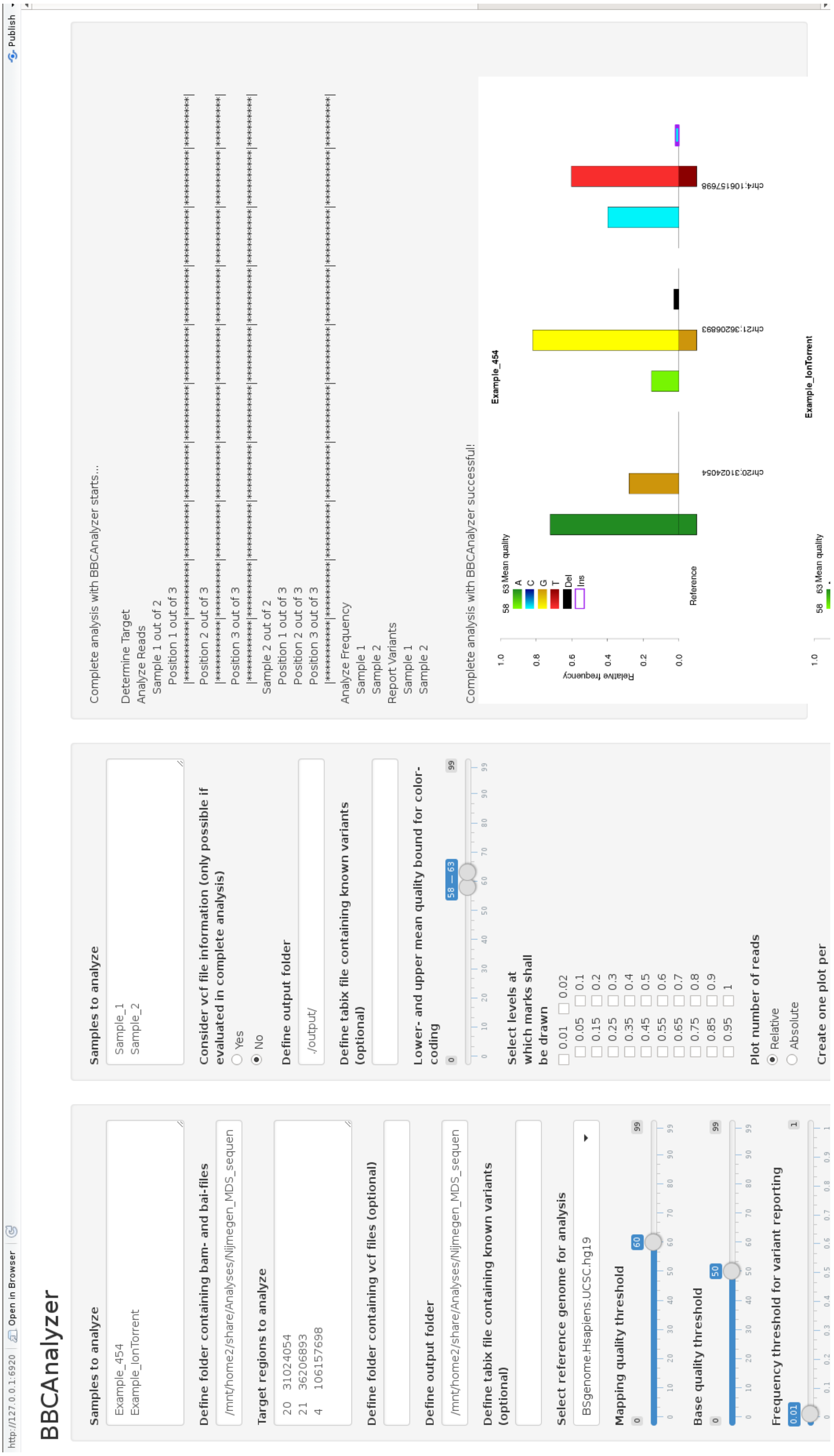

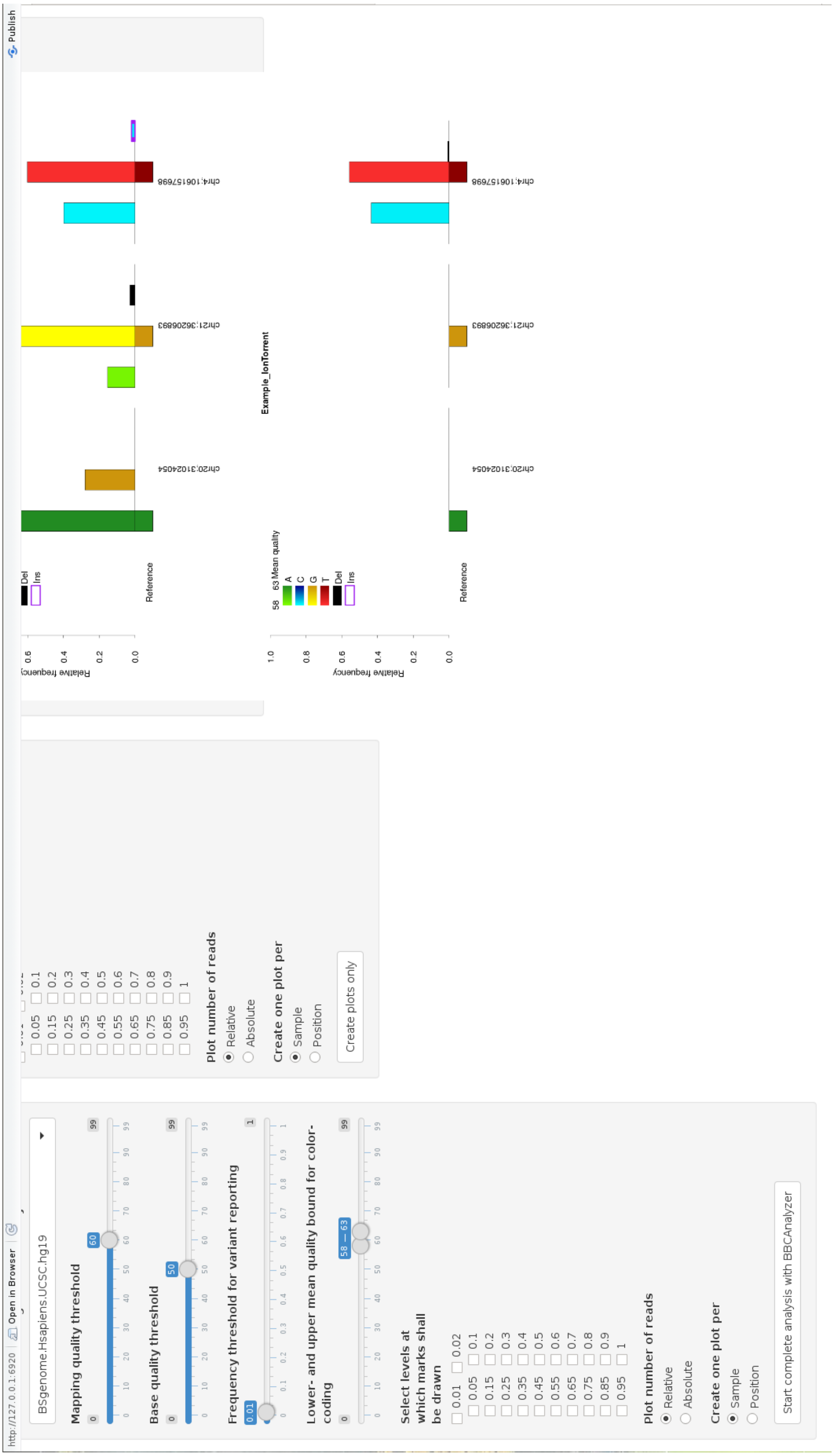

http://127.0.0.1:6920
Open in Browser
Publish
BSgenome.Hsapiens.UCSC.hg19
Mapping quality threshold
Base quality threshold
Frequency threshold for variant reporting
Lower- and upper mean quality bound for color-coding
Select levels at which marks shall be drawn
Plot number of reads
Relative
Absolute
Create one plot per
Sample
Position
Start complete analysis with BBCAnalyzer
Create plots only
Example_IonTorrent
Relative frequency
Reference
Mean quality
A
C
G
T
Del
Ins
chr20:31024054
chr21:36206893
chr4:106157698
